# An allogeneic ‘off the shelf’ therapeutic strategy for peripheral nerve tissue engineering using clinical grade human neural stem cells

**DOI:** 10.1038/s41598-018-20927-8

**Published:** 2018-02-13

**Authors:** C. O’Rourke, A. G. E. Day, C. Murray-Dunning, L. Thanabalasundaram, J. Cowan, L. Stevanato, N. Grace, G. Cameron, R. A. L. Drake, J. Sinden, J. B. Phillips

**Affiliations:** 1 Department of Biomaterials & Tissue Engineering, UCL Eastman Dental Institute, University College London, London, UK; 2 UCL Centre for Nerve Engineering, London, UK; 3 ReNeuron, Pencoed, Bridgend, Wales UK; 4 Royal National Orthopaedic Hospital, Stanmore, UK; 5 Sartorius Stedim Biotech, Royston, UK; 6 Department of Pharmacology, UCL School of Pharmacy, University College London, London, UK

## Abstract

Artificial tissues constructed from therapeutic cells offer a promising approach for improving the treatment of severe peripheral nerve injuries. In this study the effectiveness of using CTX0E03, a conditionally immortalised human neural stem cell line, as a source of allogeneic cells for constructing living artificial nerve repair tissue was tested. CTX0E03 cells were differentiated then combined with collagen to form engineered neural tissue (EngNT-CTX), stable aligned sheets of cellular hydrogel. EngNT-CTX sheets were delivered within collagen tubes to repair a 12 mm sciatic nerve injury model in athymic nude rats. Autologous nerve grafts (autografts) and empty tubes were used for comparison. After 8 weeks functional repair was assessed using electrophysiology. Further, detailed histological and electron microscopic analysis of the repaired nerves was performed. Results indicated that EngNT-CTX supported growth of neurites and vasculature through the injury site and facilitated reinnervation of the target muscle. These findings indicate for the first time that a clinically validated allogeneic neural stem cell line can be used to construct EngNT. This provides a potential ‘off the shelf’ tissue engineering solution for the treatment of nerve injury, overcoming the limitations associated with nerve autografts or the reliance on autologous cells for populating repair constructs.

## Introduction

Tissue engineering provides opportunities to combine therapeutic cells with materials in order to construct living artificial tissues that can repair nervous system injury where significant amounts of tissue have been lost. In order for this approach to be successful it is necessary to identify sources of cells that are compatible with tissue engineering technology and suitable for translation to the clinic and eventually commercial manufacture. Autologous stem cells have been explored extensively but because of variability between patients and time required for preparation there is substantial interest in the use of allogeneic cells which can be available immediately as an ‘off-the-shelf’ therapeutic product, manufactured and validated for clinical use^1^.

CTX0E03 is a clonal human neural stem cell line derived originally from foetal cortex. It is conditionally immortalised with the *c*-mycER^TAM^ transgene that generates a fusion protein that drives cell proliferation only in the presence of 4-hydroxytamoxifen (4-OHT), allowing continuous and stable cell expansion and improving safety after implantation since in the absence of 4-OHT *in vivo* the fusion protein cannot function (Pollock *et al*.^2^). The CTX cell line has been manufactured according to Good Manufacturing Practice (GMP) to ensure reliable and reproducible stocks of cells for use in clinical applications^2–4^.

The cell line is multipotent with the capacity to differentiate into neurons, astrocytes, and oligodendrocytes *in vitro* and *in vivo*^5^. Further, immunomodulation, angiogenesis and neurogenesis were observed following CTX0E03 implantation in animal models of stroke and critical limb ischemia^6,7^. Moreover, CTX0E03 is suitable for allogeneic implantation in humans without immunosuppression and was recently used in a Phase II clinical trial for patients with stable stroke disability, the PISCES trial (NCT02117635, Clinicaltrials.gov) and in a Phase I safety clinical trial to treat lower limb ischemia (NCT01916369, Clinicaltrials.gov)^5,6,8,9^. The proven suitability for clinical translation of CTX0E03 combined with its nervous system origin make CTX0E03 a promising candidate cell line for use in neural tissue engineering, particularly for the treatment of peripheral nerve injuries where there are currently no living allogeneic cellular repair options available. In addition to the advantages of being available ‘off-the-shelf’, the allogenicity of therapeutic cells such as CTX0E03 can be beneficial by promoting a paracrine effect that can induce a regenerative local tissue environment^7,10^.

Peripheral nerve injury represents a major clinical concern worldwide and causes pain and disability, with significant costs to individuals and healthcare systems. Although endogenous peripheral nerve regeneration is possible, it is a slow and often an incomplete process, particularly in larger gaps^11,12^. The clinical gold standard treatment for repair of nerve gaps more than a few centimetres remains the autograft, with alternatives limited to hollow tubular conduits or decellularised nerve allografts^13,14^. The key difference between the autograft and these other approaches is the presence of columns of living Schwann cells that are present in the autograft and provide support and guidance to regenerating neurons^15^.

Tissue engineered conduits combining therapeutic cell technologies and biomaterials are capable of providing trophic support and recreating key features of the autograft. The limited availability of autologous Schwann cells and the importance of Schwann cell phenotype and extracellular matrix architecture in supporting nerve regeneration present particular challenges in this regard. Therefore there is much interest in identifying reliable sources of therapeutic cells that can mimic the regeneration-supporting phenotype of Schwann cells^16^, and in optimising biomaterial environments to accelerate and direct axonal regeneration^11,15,17–20^.

We have developed engineered neural tissue (EngNT), an aligned sheet of cellular collagen hydrogel that can support and guide nerve regeneration *in vitro* and *in vivo*^21^. EngNT is a living replacement tissue formed using a combination of cellular self-alignment and stabilisation by plastic compression^22^. Previous studies demonstrated that EngNT could be formed using a Schwann cell line^21^, differentiated adipose derived stem cells^23^ and differentiated human dental pulp stem cells^24,25^. In each case the EngNT was able to support neuronal regeneration *in vitro* and could be delivered within a tube to a rat sciatic nerve injury site, supporting regeneration *in vivo*.

In order to progress EngNT and other cellular biomaterial approaches for nerve repair towards clinical translation, the use of CTX0E03 cells as an allogeneic ‘off the shelf’ option for peripheral nerve repair was investigated. EngNT constructs containing CTX0E03 cells (EngNT-CTX) were delivered within a NeuraGen™ conduit and implanted into a sciatic nerve repair model using athymic nude rats and tissue regeneration and restoration of function were evaluated.

## Results

### Functional reinnervation of muscle

**Figure 1:**
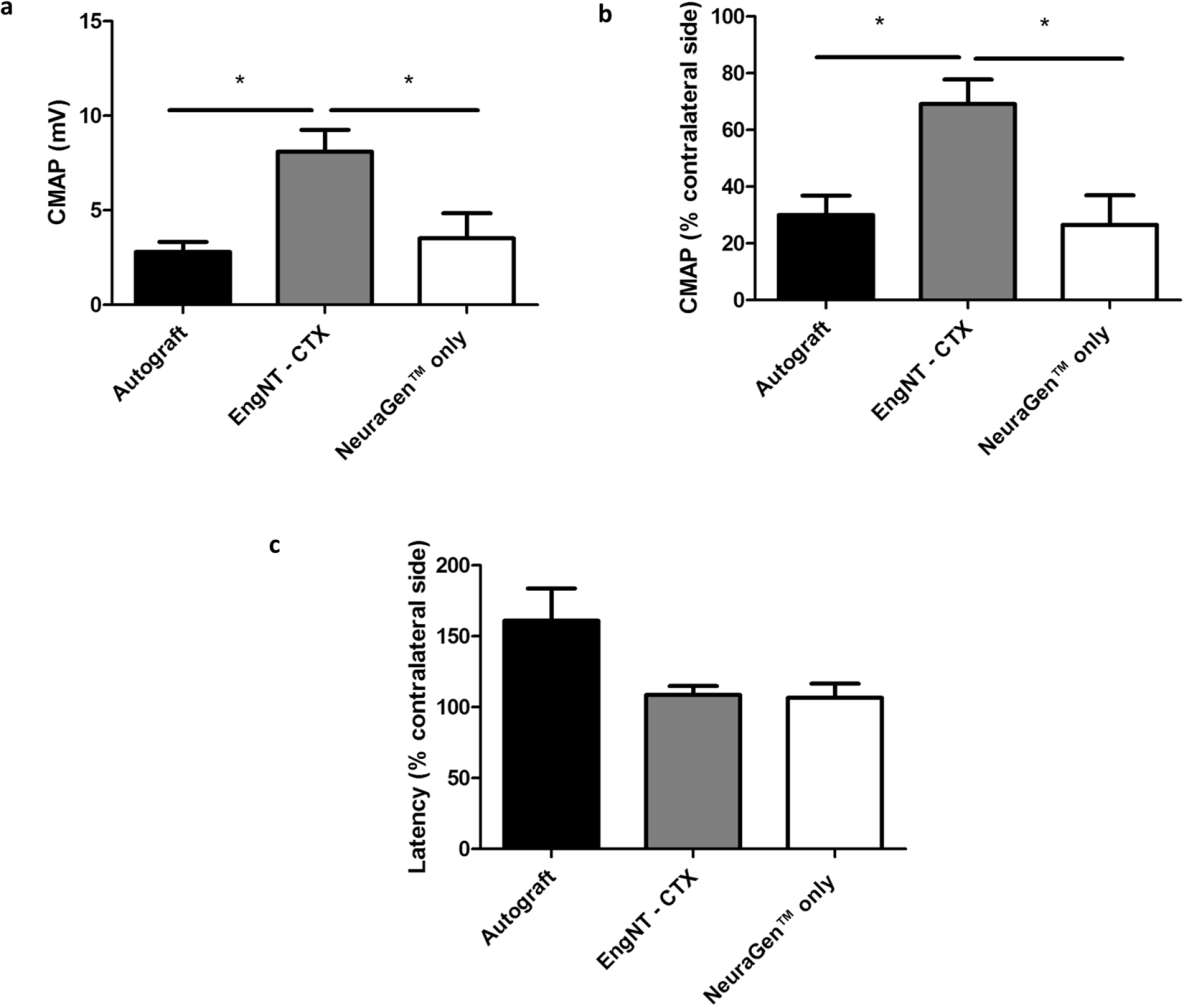
Electrophysiological evaluation of sciatic nerve 8 weeks postoperatively. Functional recovery was assessed through stimulation of the sciatic nerve proximal to the repair site and recording of CMAP in gastrocnemius muscle. CMAP amplitude values for repaired nerves (**a**) were also expressed as a percentage of the CMAP amplitude recorded from the respective contralateral control nerves (**b**) Data are means ± SEM, n = 6 (autograft), 5 (EngNT-CTX) and 4 (NeuraGen™). ANOVA with Tukey’s multiple comparisons test indicated significant differences in amplitude, *p < 0.05, **p < 0.01. (**c**) Latency associated with evoked CMAP responses in repaired nerves was expressed as a percentage of the respective contralateral value.

**Figure 2:**
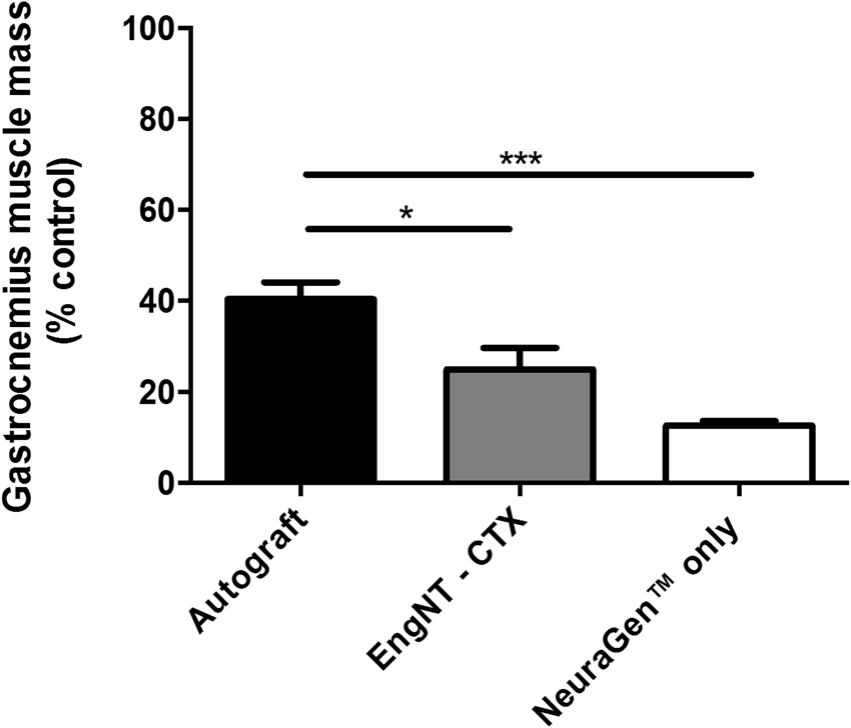
Gastrocnemius muscle mass following sciatic nerve repair. Data are means ± SEM, n = 6, of the mass of the gastrocnemius muscle on the repaired side as a % of the contralateral control muscle. ANOVA with Tukey’s multiple comparisons test, *p < 0.05, ***p < 0.001.

Differentiated CTX0E03 cells were able to self-align and undergo stabilisation to form EngNT-CTX, which was rolled into rods and placed within NeuraGen™ conduits for testing *in vivo* using athymic nude rats. To investigate functional regeneration in addition to studying the repaired tissue histologically, the muscle response to stimulation of the proximal nerve was investigated, along with the muscle mass which declines over time in the absence of innervation. Compound muscle action potentials (CMAP) were recorded from the contralateral side in all 18 animals and from the repaired side in all 6 animals in the autograft group, 5/6 in the EngNT-CTX group and 4/6 in the NeuraGen™ group (Fig. 1). In one of the EngNT samples and two of the NeuraGen™ samples CMAP amplitude was below the threshold of detection. Higher amplitudes were recorded for the CMAPs in the repairs that used EngNT-CTX compared to the other groups (Fig. 1a), with values reaching 69 ± 7%) of the contralateral uninjured side compared to autograft (30 ± 7%) and NeuraGen™ only (27 ± 10%) repairs (Fig. 1b). The latency of the evoked action potentials was greater in the repaired nerves compared with contralateral controls in all cases, with a trend for the latency values of EngNT-CTX and NeuraGen™ to be lower than the autograft, although this was not significant (Fig. 1c). Gastrocnemius muscle mass was reduced on the nerve-injured side compared to the contralateral uninjured side in all animals. Eight weeks after damage and repair the muscle mass associated with the autograft repairs was 40% of the contralateral controls, which was significantly greater than in the EngNT-CTX (24%) and NeuraGen™ only (12%) groups (Fig. 2).

### Quantification of neuronal growth

**Figure 3:**
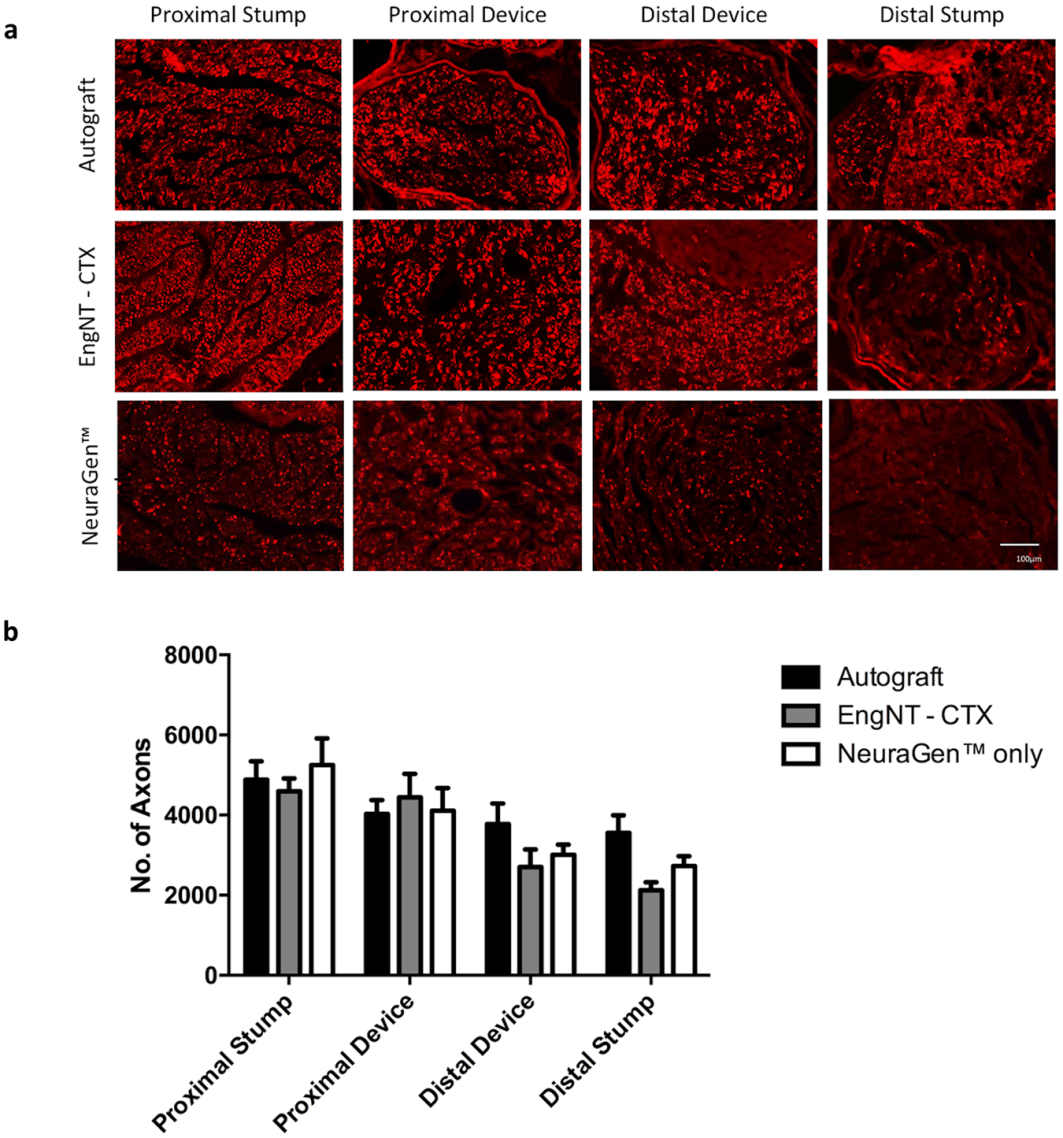
Quantification of neurites at different positions through repaired nerves. (**a**) Micrographs are transverse sections showing neurofilament positive neurites at various positions in the repaired nerves (scale bar 100 μm). Neurite growth was assessed by counting the number of neurofilament-positive axons at four different positions through the repairs, proximal stump (PS), proximal device (PD), distal device (DD) and distal stump (DS). Data are means ± SEM, n = 6, showing the number of axons in each repair at the different positions (**b**). Two-way ANOVA with Tukey’s multiple comparisons test revealed a significant difference (P < 0.0001) in neurites between the four different positions analysed, but no significant difference between the three groups in any position.

Repaired nerves were dissected and transverse sections through specific regions assessed to determine the number of neurofilament-positive neurites present in the proximal and distal stumps and proximal and distal parts within the repair site (Fig. 3a). In all groups the number of neurites counted decreased with distance distally (Fig. 3b). All groups showed similar numbers of axons in the proximal stump, proximal device and distal device, and in all cases there were neurites present in the distal stump, with a higher mean number in the autograft group compared to other conditions although this was not statistically significant.

**Figure 4:**
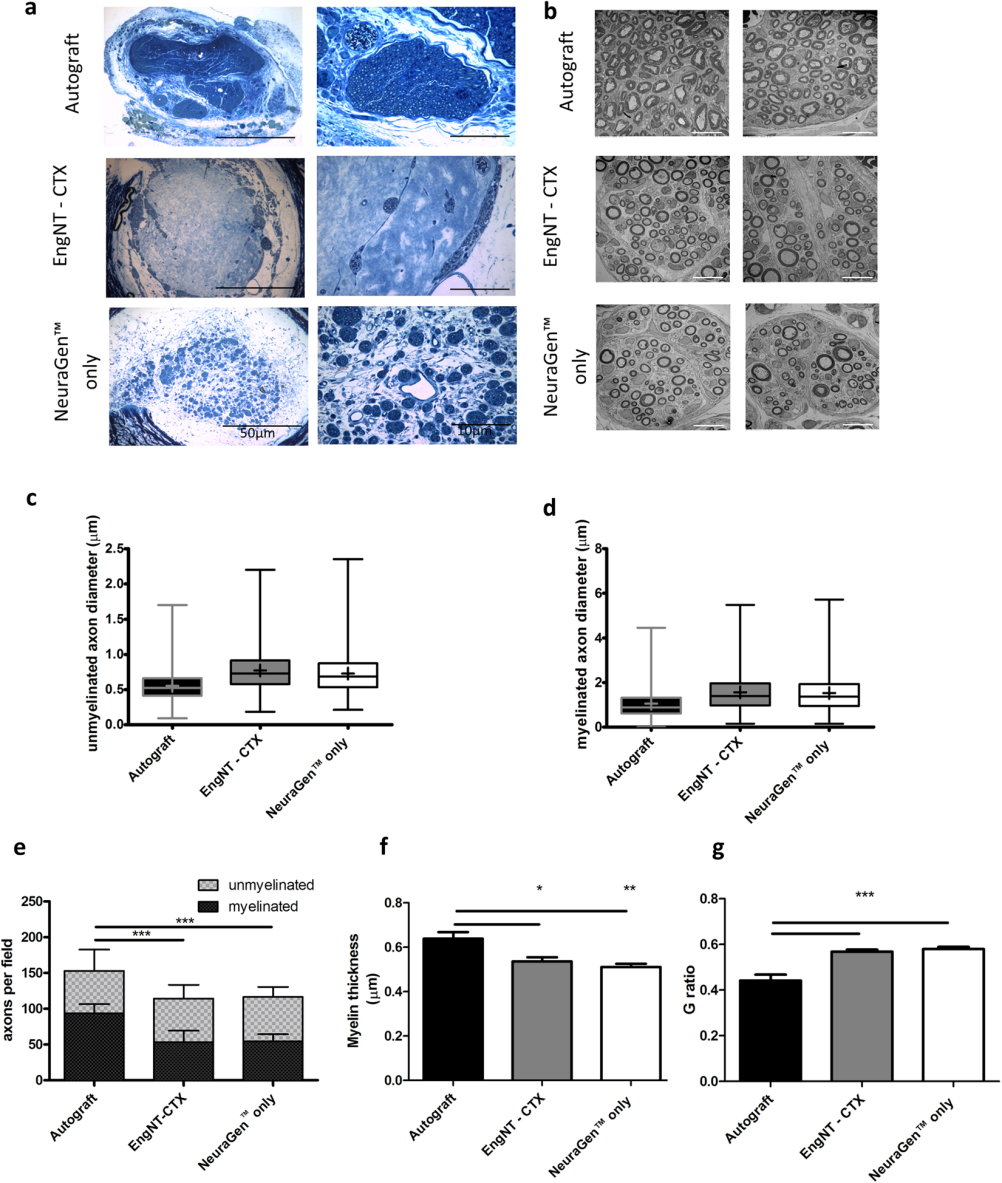
Distribution and ultrastructure of neural tissue growth at the midpoint of the repair site. Representative semi-thin sections stained with toluidine blue (**a**) show the differences in density and distribution of neural tissue growth between each condition. Five areas with the highest density in each case were sampled using TEM, with two representative images shown (**b**). Transmission electron micrographs were quantified to reveal numbers of myelinated and unmyelinated axons per 2000 µm^2^ field (**c**), with significant differences in the numbers of myelinated but not unmyelinated axons between groups. (**d**) and (**e**) are box plots showing distribution of myelinated and unmyelinated axon diameters respectively (boxes extend from the 25th to 75th percentiles, whiskers show min to max values, horizontal line indicates median and + indicates mean). For myelinated fibres the myelin thickness (**f**) and G-ratio (**g**) were also calculated. Data are means ± SEM, n = 6. ANOVA with Tukey’s multiple comparisons test *p < 0.05, **p < 0.01, ***p < 0.001.

Transverse sections were taken from the middle of the repair site and prepared for TEM. Figure 4a shows representative 0.5 μm sections stained with toluidine blue revealing the extent of regenerated nerve tissue in each group. Dense neural tissue can be observed within fascicular structures in the autograft, on and within the outer layers of the EngNT-CTX, and in patches towards the centre of the NeuraGen™. TEM was used to explore the ultrastructure of the tissue in the regions of dense neural tissue growth (Fig. 4b). The myelin sheaths appeared to be disrupted in the autograft group, possibly as a result of initial paraformaldehyde fixation being suboptimal for the preparation of TEM specimens. Some morphometric quantification was performed from the electron micrographs to enable comparison between groups, revealing a 1.7-fold increase in the number of myelinated nerve fibres present per field in autografts over the number in the other two groups (Fig. 4c). The populations of myelinated and unmyelinated axons in the autograft group included more smaller-diameter fibres compared to EngNT-CTX and NeuraGen™ (Fig. 4d and e), while myelin thickness was greater in autografts (F) with a corresponding reduction in G ratio (G).

### Macrophage presence and phenotype

**Figure 5:**
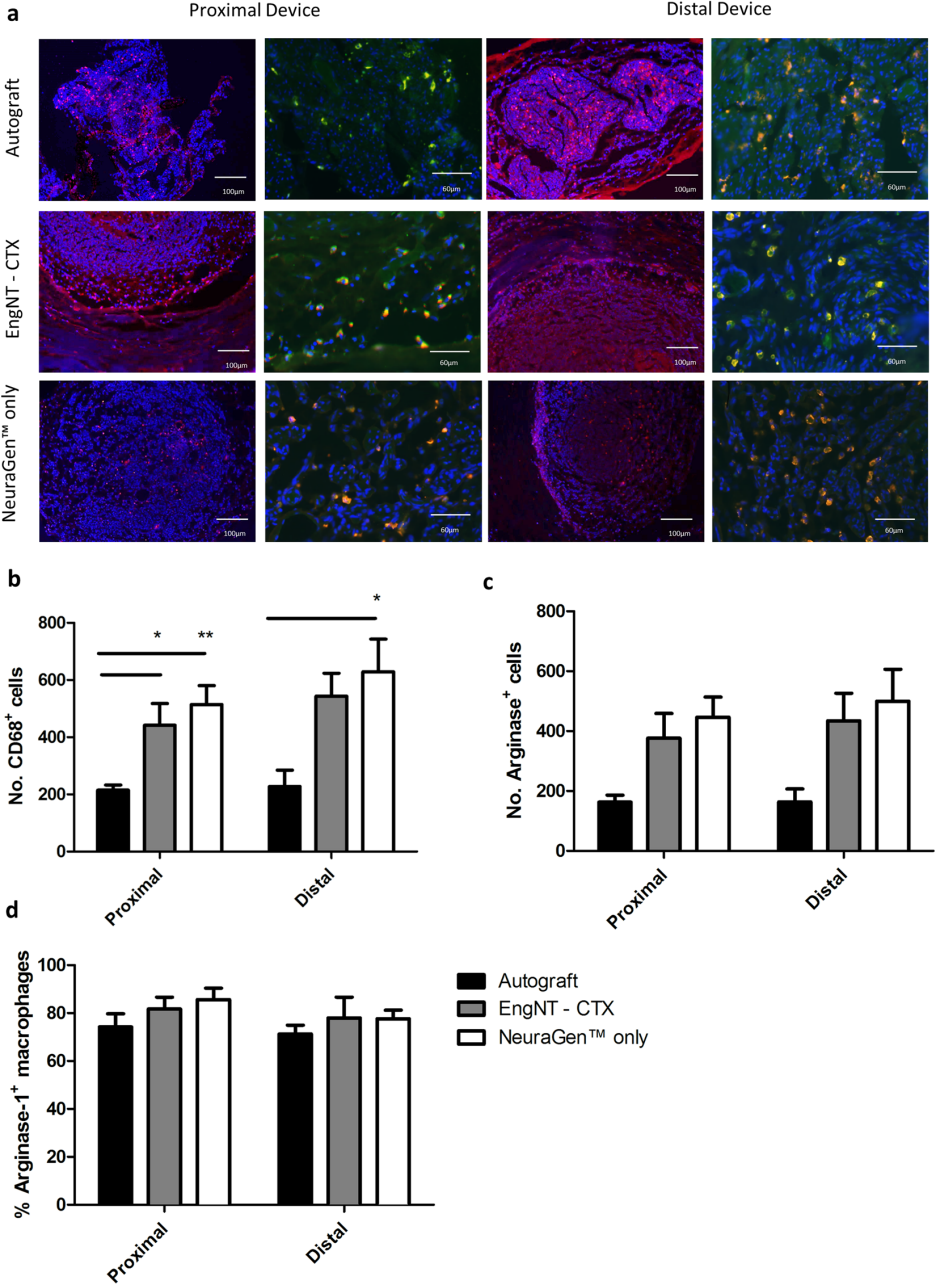
Quantification of macrophage presence and phenotype in proximal and distal parts of the repair site. Fluorescence micrographs (**a**) show the presence of CD68^+^ (red) macrophages and the arginase-1 labelled sub-population of M2 phenotype cells. Hoechst staining was included to detect cell nuclei (blue). Numbers of macrophages positive for CD68 (**b**) and arginase-1 (**c**) were quantified in each transverse section, and the proportion of CD68 labelled macrophages that also expressed arginase-1 in each region was calculated (**d**). Data are means ± SEM, n = 6. ANOVA with Tukey’s multiple comparisons test, *p < 0.05, **p < 0.05.

Immunohistochemical staining with CD68 was used to identify and quantify the total number of macrophages present in the proximal and distal parts of the repair site (Fig. 5a). Additionally, the sub-population of CD68^+^ macrophages that were also immunoreactive for arginase-1 was quantified to establish the proportion of ‘alternatively activated’ M2 phenotype macrophages compared to the ‘classically activated’ M1 cells. Samples from the EngNT-CTX group contained significantly more macrophages (CD68^+^) than the autograft in transverse sections through both the proximal (441 ± 76 versus 215 ± 18) and distal (543 ± 80 versus 227 ± 57) parts of the device (Fig. 5b). However, it is important to note that the majority of macrophages were associated with the surrounding NeuraGen™ tube rather than the EngNT-CTX material itself, and a similar distribution was observed in the NeuraGen™ only condition, with 514 ± 66 macrophages in the proximal part and 629 ± 114 in the distal. The majority of macrophages were positive for arginase-1, indicating that the macrophages present in all cases after 8 weeks exhibited a predominantly M2 phenotype (Fig. 5c and d).

### Vascularisation at the repair site

**Figure 6:**
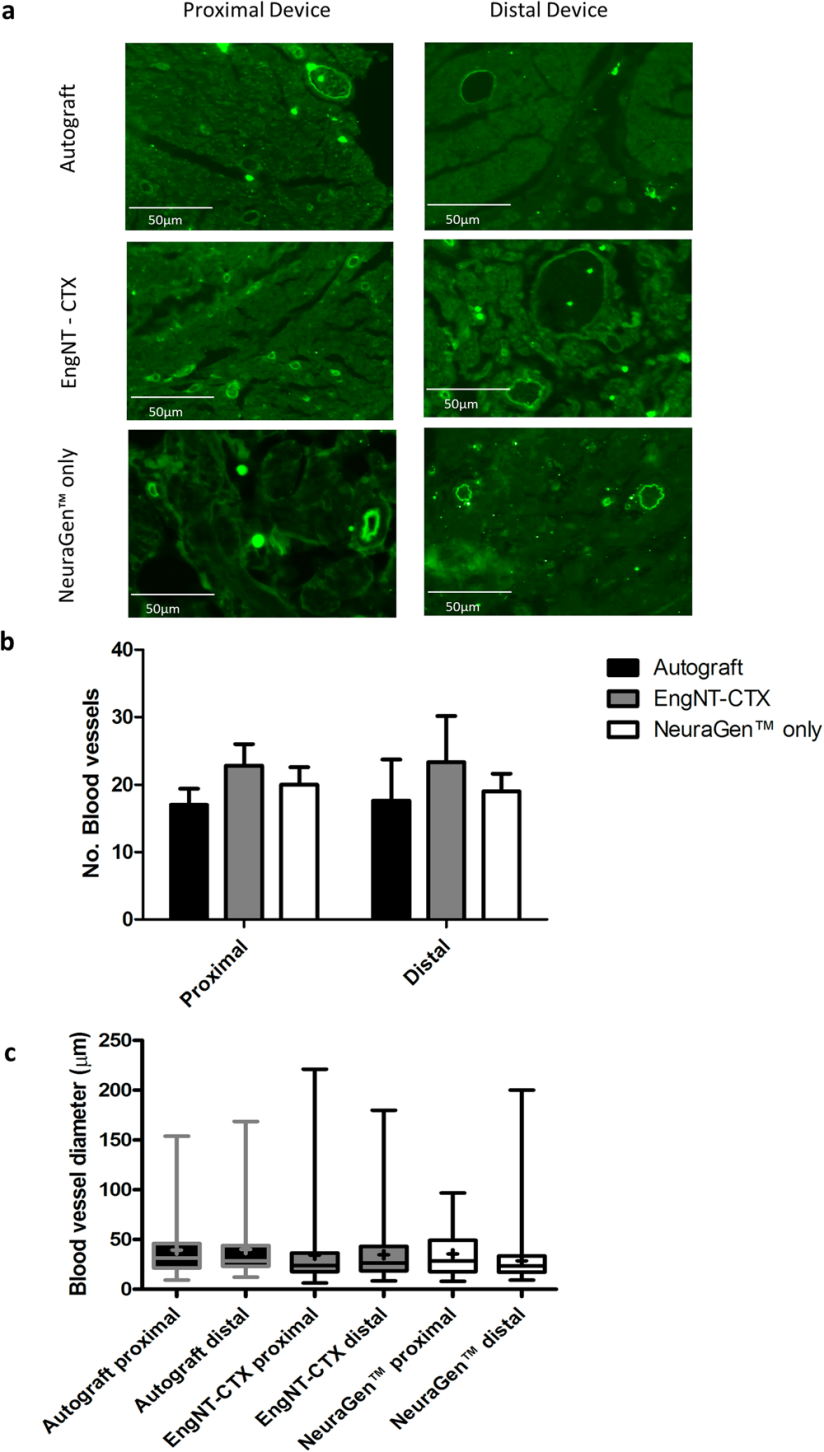
Examination of vasculature within repair site. (**a**) Quantitative analysis of number (**a**) and diameter (**b**) of blood vessels in transverse sections through the proximal and distal parts of the repair site (box plots show min and max, + indicates mean). ANOVA indicated no significant differences. Data are means ± SEM, n = 6.

The vascularisation of EngNT-CTX was examined via immunohistochemical staining of transverse sections using RECA-1 and compared to autograft and NeuraGen™ groups. Analysis revealed the presence of blood vessels throughout the repair sites in both proximal and distal sections, with no significant differences in numbers of vessels between locations and groups (Fig. 6). Vasculature in contralateral nerves was also examined, revealing approximately 21 blood vessels per nerve with a mean diameter of 36 µm.

## Discussion

The results of this study demonstrate that the clinical-grade neural stem cell line CTX0E03 can be used as a source of therapeutic cells for peripheral nerve tissue engineering. Following differentiation *in vitro*, cellular self-alignment then stabilisation in collagen gels to form EngNT-CTX, the aligned cellular material was tested in a preclinical model of nerve repair. Athymic nude rats were used for this test to ensure that any immune response to the presence of human cells in the EngNT-CTX group (which were not present in the control groups) did not confound the results. After 8 weeks recovery, electrophysiological testing indicated that EngNT-CTX constructs supported sufficient nerve regeneration to re-establish functional connections with the gastrocnemius muscle. Stimulation of the repaired nerve elicited CMAPs of greater magnitude than autograft and NeuraGen™ controls, indicating robust reinnervation of muscle. The CMAP recorded in the autograft group was lower than expected (30 ± 7% of contralateral), since previous reports using sciatic nerve autografts have shown CMAPs approximately 40–60% of the contralateral, although direct comparison is difficult since the most relevant studies also used a longer time (12 weeks) or a shorter (10 mm) gap^26,27^. We were unable to find literature reporting sciatic nerve repair studies with electrophysiological outcome measures in athymic nude rats however, so differences in this case may be underpinned by strain-specific variation. Functional reinnervation was also confirmed indirectly by investigating gastrocnemius muscle mass, which was greater in the EngNT-CTX group than the NeuraGen™ controls, although it didn’t reach the same level as the autograft. This pattern of restoration of muscle mass is similar to that seen previously in a study using athymic nude rats with a similar gap length where autograft controls resulted in approximately 50% muscle atrophy after 60 days^28^.

Histological analysis of cross sections through sequential parts of the repaired nerves showed a gradual decline in the number of neurites present from proximal to distal regions, with 60% of the number in the proximal stump present in the distal part of the EngNT-CTX device and approximately 40% reaching the distal stump. This was broadly similar in terms of neurite growth to previous EngNT constructs made using rat Schwann cells^21^ and differentiated adipose derived stem cells^23^, although the strain of rat and different length of gap prevent direct comparison. Interestingly the number of neurites in the proximal part of the EngNT-CTX device was equivalent to the number in the proximal stump, suggesting a level of proximal ingrowth considerably greater than that seen with previously tested EngNT materials^21,23,25^. There was little difference in neurite growth between the different groups, with the autograft resulting in a modest increase in the number of neurites reaching the distal stump compared to the other groups. This is in contrast to our previous studies using Sprague-Dawley rats and a 15 mm gap for 8 weeks, where there was a much larger difference between the autograft and empty conduit groups^21,23,25^. The present study therefore does not necessarily represent a ‘critical sized’ defect equivalent to a long gap in humans^21,29^.

The distribution of neuronal growth within the constructs reflects that seen in earlier studies, with neurites located predominantly on or near the surface of the EngNT construct^21,23,25^. The assembly of the constructs differed from these previous studies which used two rolled EngNT rods per conduit, instead using one EngNT-CTX sheet rolled up and anchored in place using fibrin glue. There was little neurite growth deep within the EngNT-CTX itself, which was unexpected based on the results of earlier EngNT studies where neurites were present throughout the material^21^. This indicates that optimising the way in which EngNT-CTX is organised within the outer tube could further increase regeneration. Interestingly the ultrastuctural analysis at the mid-point of the repair site indicated that there were more myelinated axons per field present in the autograft group compared to the others, but that this population of axons included a greater proportion of smaller diameter myelinated fibres compared to the EngNT-CTX and NeuraGen™ groups. This is consistent with the electrophysiological data to a certain extent, where the latency in the autograft group was longer than the other groups, which may be due to the nerve containing more neurons with a slower conduction velocity. The greater number of axons, thicker myelin and resulting lower G-ratio in the autograft group did not result in higher CMAP amplitude compared to the EngNT group, which may be due to larger numbers of non-conducting degenerating neurite sprouts present in autografts at this time point^30^. There were some unusual features in the electron micrographs for the autograft repairs, such as a more disrupted myelin structure compared to the other groups, which may indicate poor fixation or delayed/disrupted Wallerian degeneration in the autograft tissue and could mean that the apparent differences in myelin thickness and G-ratio in that group could be an experimental artefact. In general the axon diameters measured in this study were similar to those observed in another which looked at nerve fibre diameters following nerve repair in athymic nude rats^28^.

In addition to investigating reinnervation in terms of muscle function and nerve fibre growth, this study also explored the vascularisation of constructs and the presence of macrophages. These were considered to be important features in this first test of the use of CTX cells in a peripheral nerve environment since they are key indicators of suitability for future clinical use, and their combined interaction has been shown to facilitate initial Schwann cell guidance and regeneration after nerve injury^31^. In all groups there were CD68/Arginase-1 double positive cells, which is consistent with previous studies that indicate the importance of ‘alternatively activated’ M2 phenotype macrophages in nerve repair^32,33^. It is difficult to interpret the relevance of macrophage infiltration at 8 weeks since most studies that focus on this aspect have looked at a more acute macrophage response in the days to weeks immediately following repair. It was interesting to note that the increased number of macrophages present in the NeuraGen™ and EngNT-CTX groups seemed to be associated with the presence of the NeuraGen™ material, suggesting that a response to this component is responsible for the presence of the macrophages, which is consistent with previous findings^34^. Vascularisation of the EngNT-CTX was equivalent to autograft and NeuraGen™ repairs in terms of the number of blood vessels observed in transverse sections after 8 weeks, which was also similar to the number of vessels present in undamaged contralateral control nerve tissue. Vascularisation of nerve grafts is known to be important and previous studies have shown that inosculation, whereby anastomoses occurs between vessels in the graft and the host nerve tissue, is the primary method of revascularisation^35–37^. While inosculation may have occurred in the autograft group, the EngNT-CTX constructs will have been vascularised through angiogenesis. In a cellular construct for repair of long gap nerve injuries this is a vital process if the implanted cells are to survive and provide long-term regeneration support, so the presence of robust vascular growth throughout the EngNT-CTX repair site is an important observation. It remains to be investigated whether including vascular structures to mimic those present in autograft tissue might further improve EngNT-CTX function through more rapid initial vascularisation.

Overall the results of this study show for the first time that differentiated CTX0E03 cells can support repair when delivered in EngNT to a site of peripheral nerve injury in a rat model. The range of results from the functional and histological outcome measures used here indicate that further optimisation of the EngNT-CTX approach could be of benefit. Specifically, comparison with the autograft group showed scope for more neurite growth through the repair site and more effective restoration of muscle mass. Future work will focus on optimising EngNT-CTX constructs to improve these outcomes.

EngNT-CTX improved CMAP and reduced latency compared to both control groups, while gastrocnemius muscle mass and number of axons at the mid-point of the repair were lower in EngNT-CTX than in the autograft group. There were more myelinated axons present in the autograft group but they were of smaller diameter than in the other two groups. Overall the three groups showed similar numbers of neurites growing through the repair site, with similar vascularisation. There were also more macrophages present in the EngNT-CTX and NeuraGen™ groups.

The approach reported here is an important new advance since CTX0E03 is a robust clinical grade cell line which is manufactured to GMP standards and has gained regulatory approval for allogeneic cell therapy use in humans in other indications. A key advantage of cells conditionally immortalised using the *c*-mycER^TAM^ transgene is that this provides a safety switch to prevent inappropriate cell division after implantation^2^, thus overcoming potential risks associated with cell transplantation in order to facilitate regulatory approval. Furthermore, using a fully characterised clonal cell line that can be manufactured at scale overcomes the variability in performance and yield associated with autologous sources of therapeutic cells. In conclusion, EngNT-CTX provides an opportunity for ‘off the shelf’ living artificial tissue to be generated, which is suitable for commercial and clinical development as a replacement for the nerve autograft.

## Material and Methods

All experimental procedures involving animals were conducted in accordance with the UK Animals (Scientific Procedures) Act (1986)/the European Communities Council Directives (86/609/EEC) and approved by the UCL Animal Welfare and Ethics Review Board.

All data generated or analysed during this study are included in this published article (and i Supplementary dataset).

### Culture and differentiation of CTX cells

Human neural stem cells (CTX0E03, level P25-P33, ReNeuron Ltd, UK) were expanded using previously described methods^2^. CTX0E03 cells were cultured in Dulbecco’s Modified Eagles Medium:F12 medium (Gibco) supplemented with human albumin solution (0.03%; Grifols); Glutamax (2 mM; Gibco); human transferrin (5 μg/ml; Sigma), putrescine dihydrochloride (16.2 μg/ml; Sigma), human insulin (5 μg/ml; Sigma), progesterone (60 ng/ml; Sigma), sodium selenite (40 ng/ml; Sigma), epidermal growth factor (20 ng/ml; Sigma), basic fibroblast growth factor (10 ng/ml; Invitrogen), and 4-OHT (100 nM; Sigma) in 175 cm^2^ laminin-coated (20 µg/ml; Amsbio) flasks. Following expansion, CTX cells were differentiated for 2 weeks by removal of growth factors and 4-OHT. Previous work characterising the resulting cell population indicated the upregulation of neuronal and glial markers^38^ (Supplementary Figure 1).

### Fabrication of EngNT-CTX

Following differentiation, CTX cells were used to create EngNT according to methods described previously^21^. All gels were prepared using 80% v/v Type I bovine dermis collagen (3 mg/ml; Koken, diluted to 2 mg/ml using 1 mM HCl) mixed with 10% v/v 10 × minimum essential medium (Sigma) and neutralised using RAFT Neutralising Solution (Lonza Bioscience) before addition to 10% v/v CTX cell suspension to give a cell density of 2 × 10^6^ cells/ml of gel. Gels were allowed to set in tethering moulds at 37 °C for 15 min and then immersed in culture medium and incubated at 37 °C in a humidified incubator with 5% CO_2_/95% air for 24 h, during which time the cells contracted the tethered gels and become aligned^39^ (Supplementary Figure 2). Using RAFT absorbers (Lonza Bioscience) the aligned gels were stabilised for 15 minutes, a process whereby a biocompatible absorbent material is placed upon the gel and absorbs interstitial fluid to generate a dense robust hydrogel with a 50 fold increase in cell and collagen density. The resulting sheets of EngNT were rolled to form rods (12 mm length) and each construct was secured within a NeuraGen™ sheath (13 mm long) using Fibrin glue (TISSEEL, Baxter), ready for implantation.

### Surgical repair of rat sciatic nerve

Athymic nude female rats (180–200 g; Charles River) were deeply anesthetised by inhalation of isoflurane, the sciatic nerve of each animal was exposed at mid-thigh level, transected and then either a repair conduit or a nerve graft (autologous nerve tissue reversed and replaced) was positioned between the stumps to produce an inter-stump distance of 12 mm. Conduits or grafts were retained in place using 10/0 epineurial sutures at each stump, then wounds were closed in layers and animals were allowed to recover for 8 weeks. Animals were randomised to three groups (6 rats in each): (A) empty NeuraGen™, (B) EngNT-CTX in NeuraGen™ or (C) a 12 mm nerve autograft.

### Testing functional reinnervation of muscle

After 8 weeks animals were anesthetized and nerve function assessed electrophysiologically (using a Sapphire 4ME system) by comparing the repaired nerve to the contralateral undamaged nerve in each animal. A grounding electrode and a reference electrode (Ambu® Neuroline 710) were attached to the animal. A stimulating electrode (Neurosign Bipolar Probe 2 × 100 mm × 0.75 mm electrode) was placed proximal to the repair site on the surface of the nerve and a recording electrode (Ambu® Neuroline concentric) was placed into the gastrocnemius muscle. The distance between the stimulating and recording monopolar electrodes was standardized. Electrophysiological stimulation of the nerve was performed in a bipolar stimulation constant voltage configuration and muscle response recorded. Stimulation threshold was determined by increasing the stimulus amplitude in 0.1 V steps (200 µs pulse) up to 15 V, until a reproducible, stimulus-correlated muscle action potential was recorded. The latency was measured from the time of stimulus to the first deviation from the baseline, and the amplitude of the compound muscle action potential was measured from baseline to the greatest negative peak. Recordings were conducted in triplicate for the repaired nerve and contralateral control nerve in each animal. Immediately following electrophysiology analysis, animals were culled and their bilateral gastrocnemius muscles were excised and weighed.

### Histological analysis of tissue repair

Repaired nerves were excised under a dissecting microscope and immersion-fixed in 4% paraformaldehyde at 4 °C. The middle of the repair device was removed and prepared for transmission electron microscopy (TEM) and transverse cryostat sections (10 μm thick) were prepared from the remaining proximal and distal parts of the device and the nerve stumps. The transverse sections that were used for analysis were from positions 1 mm into the proximal and distal stumps, or 1 mm into the proximal and distal parts of the repair site, measured from the end of the nerve stump in each case.

Sections were adhered to glass slides (Superfrost™ Plus, Thermo Fisher Scientific) blocked using 5% goat serum for 10 min then incubated in primary antibodies (Table 1) overnight at 4 °C. All washes and dilutions were performed using immunostaining buffer (PBS containing 0.002% sodium azide and 0.2% Triton-X). After washing, sections were incubated with appropriate DyLight-conjugated secondary antibodies (1:300, Vector Laboratories, listed in Table 1) at room temperature for 45 min. Sections were mounted using VECTASHIELD HardSet mounting medium with DAPI (Vector Laboratories) and fluorescence microscopy (Zeiss Axio Lab.A1) was used to quantify axonal growth by counting all of the neurofilament positive axons present in each transverse section. The number of macrophages and those with an M2 phenotype were determined by counting ED1 and arginase positive cells respectively, and RECA-1 was used to visualise blood vessels, which were also counted. Blood vessel diameter was measured from images captured (Zeiss AxioCam) using ImageJ.

**Tablee 1:** List of antibodies used for histological analysis.

**Antibody**	**Target**	**Dilution**	**Species**	**Source**	**Secondary**
Neurofilament	Axons	1/1000	Mouse	Covance	Anti-mouse 549
CD68 (ED1)	Macrophages	1/100	Mouse	Millipore	Anti-mouse 488
Arginase	M2 Macrophages	1/200	Goat	Santa Cruz	Anti-goat 594
Reca-1	Endothelial cells	1/100	Mouse	Abcam	Anti-mouse 488

### Transmission Electron Microscopy

After excision and dissection of the middle of the repair constructs, samples that had been fixed in 4% (w/v) paraformaldehyde in PBS for 24 h were transferred to 3% glutaraldehyde (Agar Scientific) in 0.1 M cacodylate buffer. These were post-fixed in 1% (w/v) osmium tetroxide in PBS, dehydrated through a graded series of ethanol incubations, flat-embedded in TAAB embedding resin and polymerized at 60 °C for 48 h. Semi-thin sections of 0.5 μm were cut using a diamond knife on a Ultracut E microtome (Leica, UK), dried onto microscope slides and stained with 1% (w/v) toluidine blue with added 5% (w/v) sodium borate. Ultrathin sections of 70 nm were cut with a diamond knife (Diatome, UK) and collected on copper slot grids with Formvar/carbon support films. Sections were counter-stained with ethanol based uranyl acetate and Reynolds’ lead citrate before examination in a Philips CM12 TEM. Ultrathin sections were imaged at a column magnification of × 2000 from the five areas of greatest tissue density as identified from the respective stained semi-thin sections. Image J software was used to measure axon and fibre diameter, from which myelin thickness and G-ratio were calculated.

### Statistical analysis

Normality tests were performed to confirm data were normally distributed, then one-way or two-way analysis of variance tests (ANOVA) were conducted. Tukey’s multiple comparisons test was used to compare groups. For all tests, *p ≤ 0.05, **p < 0.01, and ***p < 0.001 were considered to be statistically significant.

## Electronic supplementary material

Supplementary Information
