# Supplementary material for: An allogeneic ‘off the shelf’ therapeutic strategy for peripheral nerve tissue engineering using clinical grade human neural stem cells
Source: Sci Rep. 2018 Feb 13;8:2951. doi: 10.1038/s41598-018-20927-8 (PMC5811594; doi:10.1038/s41598-018-20927-8)
Supplement: Supplementary file 1 — Supplementary Information [file 41598_2018_20927_MOESM1_ESM.pdf]

An allogeneic 'off the shelf' therapeutic strategy for peripheral nerve tissue engineering using clinical grade human neural stem cells

#C O'Rourke[1], #AGE Day[1], #C Murray-Dunning[1], L Thanabalasundaram[2], J. Cowan[3], L Stevanato[2], N Grace[4], G Cameron[4], RAL Drake[4], J Sinden[2] & *JB Phillips[1]

#*Authors contributed equally* to this work
[1]Department of Biomaterials & Tissue Engineering, UCL Eastman Dental Institute, University College London, London, UK
[2]ReNeuron, Pencoed, Bridgend, Wales
[3]Royal National Orthopaedic Hospital, Stanmore, UK
[4]Sartorius Stedim Biotech, Royston, UK

*Corresponding author: James Phillips; jb.phillips@ucl.ac.uk

Supplementary Data

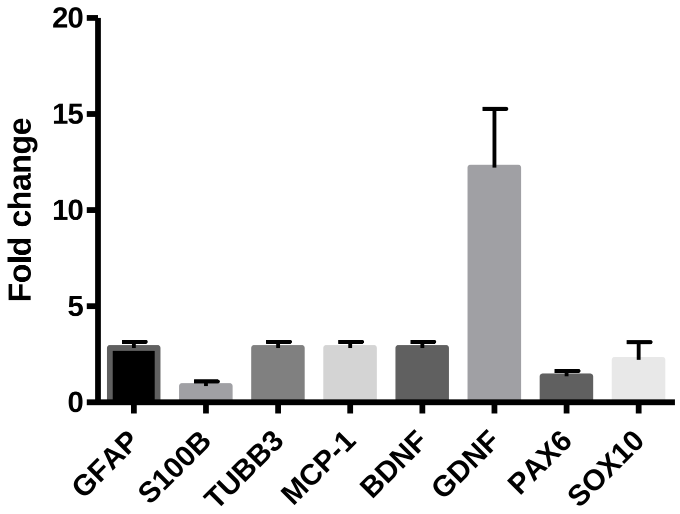

Supplementary Figure 1: Real time PCR mRNA expression levels of neuronal and glial markers in differentiated CTX cells. Cells were seeded at an initial density of 7000 cells/cm^2 and analysed after 2 weeks of differentiation (n=3; data are means ± SEM relative to undifferentiated CTX cells). Total RNA was isolated using miRNeasy (Qiagen). A minimum of 250 ng of total RNA was reverse-transcribed into first-strand cDNA using a mix of random primer and poly-dT. Reverse transcription was performed with superscript II reverse transcriptase (Invitrogen) for 1 hr at 42°C, inactivated for 15 minutes at 70°C and cooled to 4°C. Two µl of cDNA were used in a PCR reaction containing 2x Roche master mix, 0.1 µg of human universal probe library (UPL, Roche), and 0.4 µM primers for neuronal markers: βIII-tubulin (TUBB3), a well-established neuron-specific marker expressed by neuronal precursors, and glial markers: glial fibrillary acidic protein (GFAP) and S100B. The following primer sequences and UPL were used for each marker: TUBB3 (NM_006086), F) gcaactacgtgggcgact, R) cgaggcacgtacttgtgaga UPL 78; GFAP (NM_002055.3) F) ccagttgcagtccttgacct, R) ctccagggactcgttcgt, UPL 85 and S100B F) ggaaggggtgagacaagga R) ggtggaaaacgtcgatgag UPL 78. QRT-PCR results were expressed as relative quantification based on the 2-ΔΔct method and normalized against average of ATP5B and YWHAZ (PrimerDesign) housekeeping genes.

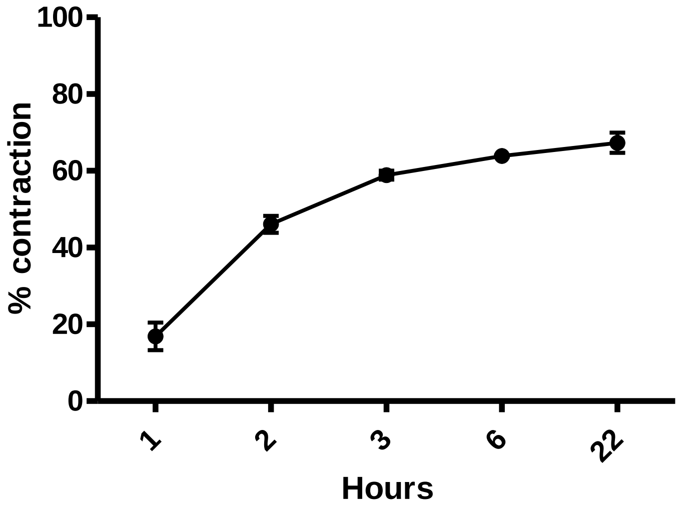

Supplementary Figure 2: Contraction of EngNT-dCTX over time. Differentiated CTX cells were set within collagen gels (2mg/ml) cast into tethering moulds at a density of $2x10^6$ per ml of collagen. Image analysis was used to calculate % contraction of the gels. Data are means ± SEM.
